# Gold Nanorod-Decorated Metallic MoS_2_ Nanosheets for Synergistic Photothermal and Photodynamic Antibacterial Therapy

**DOI:** 10.3390/nano11113064

**Published:** 2021-11-14

**Authors:** Sibidou Yougbaré, Chinmaya Mutalik, Pei-Feng Chung, Dyah Ika Krisnawati, Fajar Rinawati, Hengky Irawan, Heny Kristanto, Tsung-Rong Kuo

**Affiliations:** 1 International Ph.D. Program in Biomedical Engineering, College of Biomedical Engineering, Taipei Medical University, Taipei 11031, Taiwan; d845107003@tmu.edu.tw (S.Y.); d845108002@tmu.edu.tw (C.M.); 2 Institut de Recherche en Sciences de la Santé (IRSS-DRCO)/Nanoro, 03 B.P 7192, Ouagadougou 03, Burkina Faso; 3 School of Biomedical Engineering, College of Biomedical Engineering, Taipei Medical University, Taipei 11031, Taiwan; b812107004@tmu.edu.tw; 4 Dharma Husada Nursing Academy, Kediri 64114, Indonesia; dyah_ika_k@adhkediri.ac.id (D.I.K.); fajar_rinawati@adhkediri.ac.id (F.R.); hengky_irawan@adhkediri.ac.id (H.I.); heny_kristanto@adhkediri.ac.id (H.K.); 5 Graduate Institute of Nanomedicine and Medical Engineering, College of Biomedical Engineering, Taipei Medical University, Taipei 11031, Taiwan

**Keywords:** antimicrobial activity, gold nanorods, metallic molybdenum disulfide, phototherapy, photothermal therapy, photodynamic therapy, synergistic effect

## Abstract

Light-responsive nanocomposites have become increasingly attractive in the biomedical field for antibacterial applications. Visible-light-activated metallic molybdenum disulfide nanosheets (1T-MoS_2_ NSs) and plasmonic gold nanorods (AuNRs) with absorption at a wavelength of 808 nm were synthesized. AuNR nanocomposites decorated onto 1T-MoS_2_ NSs (MoS_2_@AuNRs) were successfully prepared by electrostatic adsorption for phototherapy applications. Based on the photothermal effect, the solution temperature of the MoS_2_@AuNR nanocomposites increased from 25 to 66.7 °C after 808 nm near-infrared (NIR) laser irradiation for 10 min. For the photodynamic effect, the MoS_2_@AuNR nanocomposites generated reactive oxygen species (ROS) under visible light irradiation. Photothermal therapy and photodynamic therapy of MoS_2_@AuNRs were confirmed against *E. coli* by agar plate counts. Most importantly, the combination of photothermal therapy and photodynamic therapy from the MoS_2_@AuNR nanocomposites revealed higher antibacterial activity than photothermal or photodynamic therapy alone. The light-activated MoS_2_@AuNR nanocomposites exhibited a remarkable synergistic effect of photothermal therapy and photodynamic therapy, which provides an alternative approach to fight bacterial infections.

## 1. Introduction

Photoresponsive nanomaterials have been extensively applied for energy conversion, electronic devices, and medical therapies [1,2,3,4,5,6,7,8]. Recently, the use of light-activated nanomaterials has been focused on phototherapy [9,10,11,12,13,14,15,16]. Phototherapy is defined as the use of photoresponsive nanomaterials to generate heat or noxious components to kill microorganisms or cells [17,18,19]. For phototherapy, there are two main approaches: photothermal therapy and photodynamic therapy [20,21,22]. Photothermal therapy is a treatment that utilizes photothermal agents activated by light irradiation. The absorbed light energy is transformed into heat by photothermal agents for therapeutic purposes [23,24,25]. Photothermal therapy was reported to induce no bacterial resistance, and therefore, it is effective against superbugs [26]. With photodynamic therapy, light-activated nanomaterials absorb light to facilitate the generation of reactive oxygen species (ROS) [27,28,29,30]. Light-induced ROS production by nanomaterials can destroy bacteria via two main mechanisms. First, ROS can bind to bacterial membranes, inducing destruction of bacterial cell walls. Second, ROS can penetrate bacterial cells to bind to and damage lipids and proteins and thus disrupt cell physiological activities, leading to bacterial death [31]. Although light-activated nanomaterials have been demonstrated for both photothermal therapy and photodynamic therapy, designing nanomaterials to provide a synergistic effect of photothermal therapy and photodynamic therapy is still an emerging task in medical therapeutics.

Different photothermal agents such as metals, metal oxides, and carbon nanomaterials have been investigated for photothermal therapy [32,33,34]. For instance, gold nanorods (AuNRs) and gold nanobipyramids were verified to be photothermal agents with highly efficient antibacterial activities [35]. A nanocomposite composed by polyurethane, polyethylene glycol, and AuNRs was prepared as a near-infrared (NIR)-responsive organic/inorganic hybrid to fight multidrug-resistant (MDR) bacteria [36]. Polypyrrole coated with Fe_3_O_4_ nanocomposites was demonstrated to increase the temperature of tumors from 32.8 to 48.8 °C within 5 min under 808 nm NIR laser irradiation [37]. Polypyrrole-based photothermal nanoantibiotics with robust photothermal effects in the NIR-II region (~1064 nm) were designed for valid treatment of MDR bacterial infections [38]. Among these photothermal agents, plasmonic metal nanomaterials have been intensively investigated because of their superior photothermal effects. More interestingly, photothermal therapy has been combined with other therapies such as photodynamic therapy, chemotherapy, radiotherapy, and so on for better therapeutic outcomes.

Various light-activated nanomaterials are popularly applied as antibacterial agents based on photodynamic therapy [39,40,41,42,43,44,45]. For example, metallic molybdenum disulfide (1T-MoS_2_) and semiconducting molybdenum disulfide (2H-MoS_2_) nanoflowers were validated to have bactericidal effects due to their light-driven ROS production [46]. A nanocomposite of iron disulfide nanoparticles (NPs) conjugated with titanium dioxide NPs was designed as a photodynamic antibacterial agent with broad absorption from the visible (Vis) to NIR region [47]. A nanocomposite of zinc oxide NPs doped with selenium was fabricated as an antibacterial nanomedicine to provide ROS to inhibit the growth of *S. aureus* under visible light illumination [48]. Graphene oxide–cuprous oxide heterostructured nanocomposites were synthesized with long-term antibacterial activity against *E. coli* and *S. aureus*; they utilized the synergistic effect of light-induced ROS production and the sustained release of copper ions [49]. Currently, great achievements have been made in applications of light-activated nanomaterials for photodynamic therapy. However, to enhance therapeutic efficiencies, nanocomposites with a synergistic effect of photothermal therapy and photodynamic therapy are still being developed.

In this work, a seed mediation approach was applied to prepare plasmonic AuNRs. Nanosheets (NSs) of 1T-MoS_2_ were prepared via a facile solvothermal method. Afterward, AuNRs were decorated onto the 1T-MoS_2_ NSs (MoS_2_@AuNRs) by electrostatic adsorption for use as an antibacterial agent in a phototherapy application. AuNRs, 1T-MoS_2_ NSs, and MoS_2_@AuNRs were characterized by ultraviolet (UV)–Vis spectroscopy, transmission electron microscopy (TEM), zeta potential, Fourier transform infrared (FTIR) spectroscopy, and powder X-ray diffraction (XRD) to confirm their optical and structural properties. The photothermal effect and photodynamic effect of MoS_2_@AuNRs were respectively investigated using an NIR laser and visible light irradiation. The photothermal therapy and photodynamic therapy of MoS_2_@AuNRs were separately examined against *E. coli* by agar plate counts. The synergistic effect of photothermal therapy and photodynamic therapy from MoS_2_@AuNRs was also evaluated as an antibacterial application.

## 2. Methods

### 2.1. Chemicals

Hexadecyltrimethylammonium bromide (CTAB, ≥98.0%), hydrogen tetrachloroaurate(III) (HAuCl_4_·3H_2_O), sodium borohydride (NaBH_4_), oleic acid (≥99%), silver nitrate (AgNO_3_), L-ascorbic acid (99%), a hydrochloric acid solution (HCl, 12 M), thiourea (>99%), molybdic acid (≥85.0% MoO_3_ basis), 2′,7′-dichlorofluorescein diacetate (H2DCFDA), and ethanol were purchased from Sigma-Aldrich (St. Louis, MO, USA). Phosphate-buffered saline (PBS; 1×, pH 7.4) was purchased from Bioshop (Burlington, ON, Canada). All chemicals were purchased and used without further purification.

### 2.2. Synthesis of AuNRs via a Seed-Mediation Method

AuNRs were prepared according to a seed-mediation method used in our previous work [35]. For the seed-mediation method, a seed solution and growth solution were first prepared. The seed solution was prepared by adding 100 µL of a HAuCl_4_ (25 mM) aqueous solution to 10 mL of a CTAB aqueous solution (0.1 M) with stirring at 30 °C. Afterward, 2 mL of a fresh, ice-cold NaBH_4_ (6 mM) aqueous solution was added to the seed solution, and then the seed solution was further stirred for 2 min. Before being added to the growth solution, the seed solution was stored for 2 h at room temperature in the dark. The growth solution (25 mL) was composed by CTAB (0.2 M) and oleic acid (0.035 M). Furthermore, 4.8 mL of an AgNO_3_ aqueous solution (4 mM) was added to the growth solution with stirring for 15 min, and then a HAuCl_4_ aqueous solution (50 mL, 1 mM) was also poured into the growth solution with stirring at 30 °C for 90 min. Afterward, the colorless growth solution was added to the HCl solution (190 µL, 12 M) for further reaction for 15 min. Finally, the growth solution was added to an ascorbic acid aqueous solution (310 µL, 0.1 M) with stirring. To synthesize AuNRs, the seed solution (35 µL) was injected into growth solution with stirring. Subsequently, the mixture of seed solution and growth solution was placed for AuNR growth overnight. For purification, the rust-red AuNR solution was centrifuged at 6000 rpm for 10 min. After centrifugation, the supernatant was carefully poured out, and the AuNR precipitate was redispersed in the CTAB solution (2 mM).

### 2.3. Preparation of 1T-MoS_2_ NSs

NSs of 1T-MoS_2_ were prepared according to a solvothermal method with some modifications [46]. Typically, a precursor solution (40 mL) containing thiourea (12.5 mM) and molybdic acid (5 mM) was prepared with stirring. After stirring for 30 min, 100 mL of the precursor solution was transferred to a PTFE-lined stainless-steel autoclave. Afterward, the autoclave was sealed and heated to 180 °C in an oven. After reacting for 24 h, the autoclave reactor was cooled to room temperature. Eventually, 1T-MoS_2_ NSs were prepared. For further application, 1T-MoS_2_ NSs were washed with water and ethanol several times and then dried at 60 °C in an oven overnight to produce black powder.

### 2.4. Synthesis of AuNR-Decorated 1T-MoS_2_ NSs

A 1T-MoS_2_ NS aqueous solution at a concentration of 100 µg/mL was prepared. AuNR aqueous solutions of three different concentrations of 100, 50, and 33.3 µg/mL were prepared in CTAB aqueous solutions (125 µM). To prepare AuNR-decorated 1T-MoS_2_ NSs, three samples of the 1T-MoS_2_ NS solution (500 µL, 100 µg/mL) were introduced into 500 µL of AuNR solutions with various concentrations of 100 µg/mL (MoS_2_@AuNRs), 50 µg/mL (MoS_2_@1/2AuNRs), and 33.3 µg/mL (MoS_2_@1/3AuNRs). The three solutions, one each of MoS_2_@AuNRs, MoS_2_@1/2AuNRs, and MoS_2_@1/3AuNRs, were then mixed at 26 °C with shaking for 3 h. After 3 h, the three solutions were centrifuged at 5000 rpm for 10 min, the supernatants were discarded, and the precipitates were further dissolved in 500 µL of a PBS solution by sonication for 5 min. Samples of MoS_2_@AuNR, MoS_2_@1/2AuNR, and MoS_2_@1/3AuNR solutions were stored at 4 °C until being used in subsequent experiments.

### 2.5. Photothermal Performance of MoS_2_@AuNRs

Photothermal performances were investigated using an 808 nm NIR laser (DPSSL DRIVER II) (ONSET, New Taipei City, Taiwan) with a power intensity of 1 W/cm^2^. Three samples, one each of MoS_2_@AuNRs, MoS_2_@1/2AuNRs, and MoS_2_@1/3AuNRs (400 µL), were separately added into microplate wells. Each sample was irradiated for 10 min, and the sample temperature was measured every minute with a thermal imaging camera during 808 nm NIR laser irradiation.

### 2.6. Evaluation of ROS Generation by MoS_2_@AuNRs

ROS generation was evaluated with a dichlorofluorescein assay. In the presence of ROS, 2′,7′-dichlorodihydrofluorescein diacetate (H2DCFDA) was oxidized to form 2′,7′-dichlorofluorescein (DCF). H2DCFDA does not fluoresce, while DCF does. The fluorescence intensity of DCF was quantitatively detected by fluorescence spectroscopy using excitation/emission at 488/525 nm. The fluorescence intensity of DCF was proportional to the concentration of ROS production. The working solution was obtained by means of PBS and two intermediate solutions named solutions I and II. Solution I was prepared by adding 4.87 mg of H2DCFDA to 10 mL of ethanol (99.5%). Sodium hydroxide (0.01 mM) was prepared as solution II. The working solution was a mixture of 10 mL of PBS, 2 mL of solution I, and 2 mL of solution II, and it was incubated for 30 min while being protected from light. After incubation, the solution was ready to use or could be stored at 4 °C. The total ROS generated were evaluated by the fluorescence intensity developed by a mixture of 1 mL of the working solution and 200 µL of the sample.

### 2.7. Antibacterial Phototherapy of E. coli

The effect of antimicrobial phototherapy of MoS_2_@AuNRs on *E. coli* was evaluated using the agar plate counting method. Briefly, three samples, one each of MoS_2_@AuNRs, MoS_2_@1/2AuNRs, and MoS_2_@1/3AuNRs (250 µL), were respectively added to 250 µL of an *E. coli* suspension with an optical density at 600 nm (OD_600_) of 0.2. These three mixtures separately underwent three types of light exposure, including an 808 nm NIR laser at a power density at 1.0 W/cm^2^, visible light, and both. Simulated solar AM1.5 light (xenon lamp, Enlitech LH150) (Enlitech, Kaohsiung City, Taiwan) was applied as the visible light source. After light exposure, these three mixtures were further cultured at 37 °C for 3 h. Afterward, 20 µL of each mixture was cultured on LB agar plates at 37 °C for 18 h.

## 3. Results and Discussion

### 3.1. Optical Properties of MoS_2_@AuNRs

**Figure 1:**
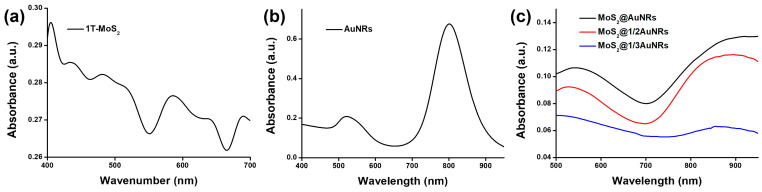
UV–Vis absorption spectra of (**a**) metallic molybdenum disulfide (1T-MoS_2_) nanosheets (NSs), (**b**) gold nanorods (AuNRs), (**c**) MoS_2_@1/3AuNRs, MoS_2_@1/2AuNRs, and MoS_2_@AuNRs.

The optical properties of 1T-MoS_2_ NSs, AuNRs, and MoS_2_@AuNRs were first characterized by UV–Vis spectroscopy. In the UV–Vis spectrum shown in Figure 1a, 1T-MoS_2_ NSs revealed two excitonic absorption bands located at wavelengths of 438 and 588 nm, corresponding to the metallic phase of MoS_2_ [50,51]. As shown in Figure 1b, the UV–Vis spectrum of the AuNRs exhibited transverse and longitudinal surface plasmon resonance (SPR) bands with maximal absorptions at ~520 and ~808 nm, respectively. For AuNRs decorated with 1T-MoS_2_ NSs, the UV–Vis absorption spectra of the nanocomposites, including MoS_2_@1/3AuNRs, MoS_2_@1/2AuNRs, and MoS_2_@AuNRs, revealed a noticeable red-shift of the longitudinal SPR band compared to that of the AuNRs, as shown in Figure 1c. The redshifts of the longitudinal SPR band for the MoS_2_@1/3AuNRs, MoS_2_@1/2AuNRs, and MoS_2_@AuNRs were 67, 101, and 117 nm, respectively. Furthermore, band broadening of the longitudinal SPR bands of AuNRs was also observed in MoS_2_@1/3AuNRs, MoS_2_@1/2AuNRs, and MoS_2_@AuNRs. The red-shift and broadening of the longitudinal SPR band of AuNRs in MoS_2_@1/3AuNRs, MoS_2_@1/2AuNRs, and MoS_2_@AuNRs could be attributed to changes in the surface refractive index of the AuNRs after decoration with 1T-MoS_2_ NSs [52]. Although the red-shift and broadening of the longitudinal SPR band were observed, the MoS_2_@1/3AuNR, MoS_2_@1/2AuNR, and MoS_2_@AuNR nanocomposites still clearly exhibited absorption at 808 nm. The absorption intensities of the MoS_2_@1/3AuNRs, MoS_2_@1/2AuNRs, and MoS_2_@AuNRs were 0.059, 0.105, and 0.113, respectively, at 808 nm. With absorption at 808 nm, the MoS_2_@1/3AuNR, MoS_2_@1/2AuNR, and MoS_2_@AuNR nanocomposites were further applied as photothermal agents.

### 3.2. Structural Characterization of MoS_2_@AuNRs

**Figure 2:**
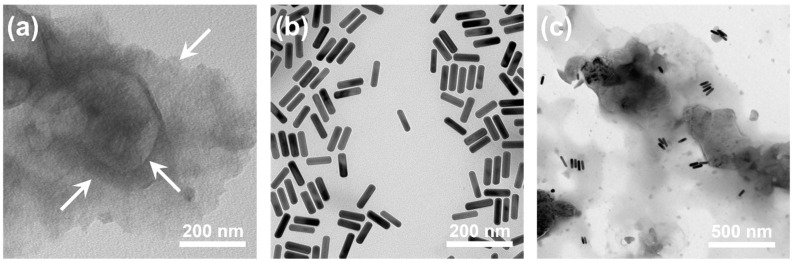
TEM images of (**a**) metallic molybdenum disulfide (1T-MoS_2_) nanosheets (NSs), (**b**) gold nanorods (AuNRs), and (**c**) MoS_2_@AuNRs.

The morphologies of 1T-MoS_2_ NSs, AuNRs, and MoS_2_@AuNRs were examined on TEM images. In the TEM image shown in Figure 2a, the NS structure of 1T-MoS_2_ NSs can be observed. The white arrows indicate different thin layers of the 1T-MoS_2_ NSs. As shown in Figure 2b, the aspect ratio (length/diameter) of the AuNRs was calculated to be 4.32, according to the average length (80.7 nm) and diameter (18.7 nm). After calculating based on the AuNRs in the TEM image, the synthetic yield we obtained exceeded 95%. As shown in Figure 2c, AuNRs were randomly decorated on the surface of 1T-MoS_2_ NSs. The zeta potentials of 1T-MoS_2_ NSs and AuNRs were negative (–32.7 mV) and positive (44.6 mV), respectively. Therefore, for MoS_2_@AuNRs, the decoration of AuNRs on 1T-MoS_2_ NSs could be attributed to electrostatic adsorption [53,54]. Overall, the results of TEM characterization indicated that MoS_2_@AuNRs were successfully prepared by electrostatic adsorption between AuNRs and 1T-MoS_2_ NSs.

### 3.3. FTIR Characterization of MoS_2_@AuNRs

**Figure 3:**
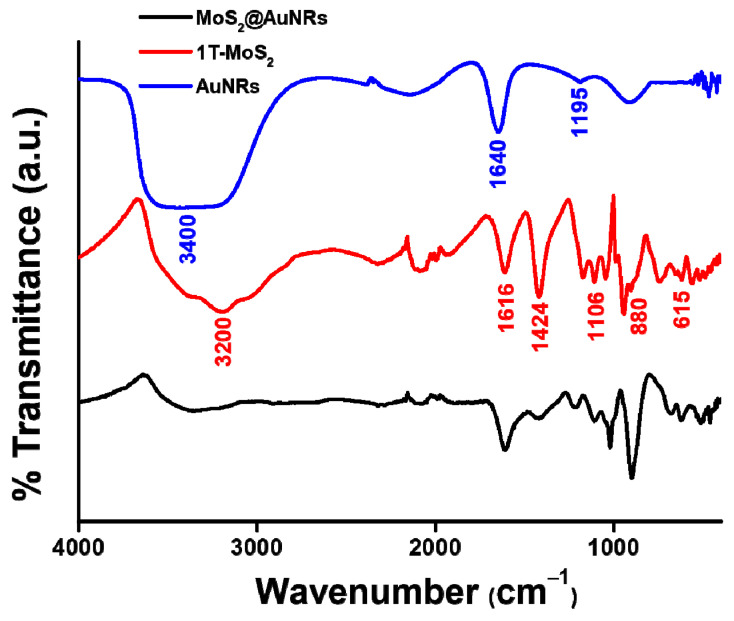
FTIR spectra of gold nanorods (AuNRs), metallic molybdenum disulfide (1T-MoS_2_) nanosheets (NSs), and MoS_2_@AuNRs.

The anchoring of AuNRs onto 1T-MoS_2_ NSs was also investigated by FTIR from 400 to 4000 cm^−1^. First, the FTIR spectra of 1T-MoS_2_ NSs and AuNRs were examined and compared to that of MoS_2_@AuNRs. As shown in Figure 3, the FTIR spectrum of 1T-MoS_2_ NSs revealed a broad band at 3200 cm^−1^ because of OH stretching of hydrogen bonds [55]. Characteristic peaks at 1616, 1424, 1106, 880, and 615 cm^−1^ were ascribed to 1T-MoS_2_ NSs. For the FTIR spectrum of AuNRs, the strong, broad band at 3400 cm^−1^ was provided by OH stretching of the adsorbed H_2_O [56]. A bending band of H_2_O was observed at 1640 cm^−1^. Moreover, the weak band at 1195 cm^−1^ was indexed as CN stretching of CTAB. The FTIR spectrum of AuNRs indicated that the CTAB surfactant was conjugated onto the surface of AuNRs. Most importantly, characteristic FTIR peaks of 1T-MoS_2_ NSs and AuNRs were observed in the FTIR spectrum of MoS_2_@AuNRs. The FTIR characterization indicated that there were no significant changes in 1T-MoS_2_ NSs or AuNRs after their decoration.

### 3.4. XRD Investigation of MoS_2_@AuNRs

**Figure 4:**
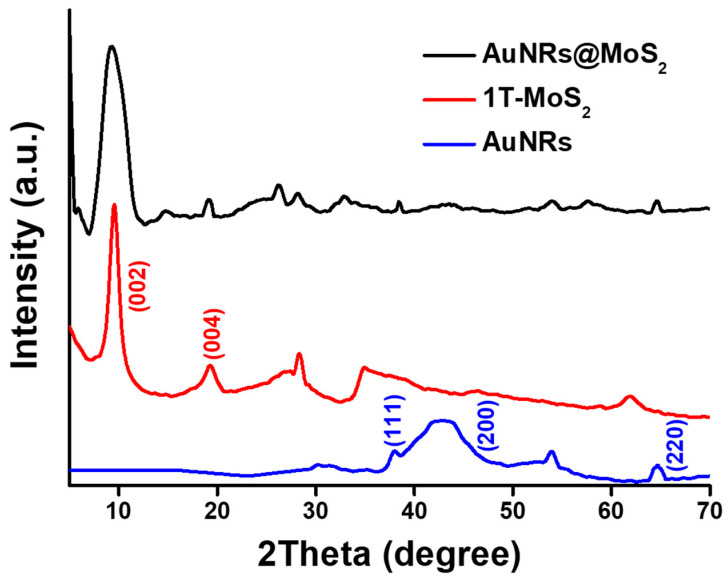
XRD spectra of gold nanorods (AuNRs), metallic molybdenum disulfide (1T-MoS_2_) nanosheets (NSs), and MoS_2_@AuNRs.

XRD spectra were measured to identify the crystallinity of the nanocomposites, including AuNRs, 1T-MoS_2_ NSs, and MoS_2_@AuNRs. As shown in Figure 4, the XRD spectrum of 1T-MoS_2_ NSs revealed two critical peaks at 9.6° and 19.1°, corresponding to the crystal planes of (002) and (004) of the 1T-MoS_2_ NSs (JCPDS No. 37-1492), respectively [57]. The XRD pattern of AuNRs revealed three main XRD peaks located at 38.2°, 44.4°, and 64.6°, corresponding to the crystal planes of (111), (200), and (220), respectively [58,59]. For MoS_2_@AuNRs, characteristic peaks of 1T-MoS_2_ NSs and AuNRs were still exhibited after the decoration of AuNRs onto 1T-MoS_2_ NSs. Results of the XRD investigation demonstrated that the crystal structures of 1T-MoS_2_ NSs and AuNRs were retained in the MoS_2_@AuNR nanocomposite.

### 3.5. Photothermal Performance of MoS_2_@AuNRs

**Figure 5:**
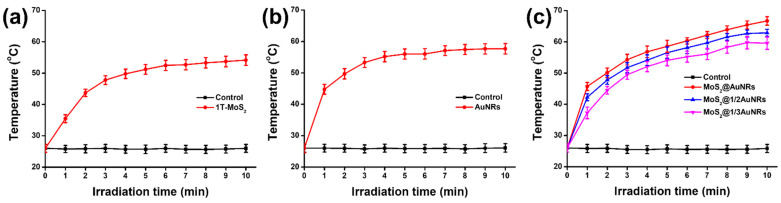
Photothermal performances of (**a**) metallic molybdenum disulfide (1T-MoS_2_) nanosheets (NSs), (**b**) gold nanorods (AuNRs), and (**c**) MoS_2_@AuNRs, MoS_2_@1/2AuNRs, and MoS_2_@1/3AuNRs. Sterilized water, CTAB aqueous solution (125 µM), and PBS solution were applied as the controls in (**a**), (**b**), and (**c**), respectively. All data are presented as the mean ± SD, *n* = 3 per group.

To compare photothermal performances, 1T-MoS_2_ NSs, AuNRs, MoS_2_@AuNRs, MoS_2_@1/2AuNRs, and MoS_2_@1/3AuNRs were illuminated by an 808 nm NIR laser. For the control experiments, the temperature of sterilized water revealed no significant increase under NIR laser irradiation, as shown in Figure 5a. After NIR laser irradiation for 10 min, the temperature of the 1T-MoS_2_ NS solution (100 µg/mL) increased from 25.0 to 54.2 °C. Moreover, as shown in Figure 5b, the temperature of the control (CTAB aqueous solution, 125 µM) showed no obvious increase under NIR laser irradiation. After NIR laser irradiation for 10 min, the temperature of the AuNR solution (100 µg/mL) increased from 25.0 to 57.6 °C. As shown in Figure 5c, the temperature of the control (PBS solution) indicated no significant increase under NIR laser irradiation. Furthermore, the final temperatures of the MoS_2_@AuNRs, MoS_2_@1/2AuNRs, and MoS_2_@1/3AuNRs were 66.7, 62.9, and 59.5 °C, respectively, after NIR laser illumination for 10 min. The results of the photothermal assays demonstrated that the photothermal performance of MoS_2_@AuNR nanocomposites increased with the concentration of AuNRs. Most importantly, with the decoration of AuNRs onto 1T-MoS_2_ NSs, MoS_2_@AuNR nanocomposites exhibited higher photothermal performance that that of 1T-MoS_2_ NSs or AuNRs. The reason could be attributed to the synergistic photothermal effect between the 1T-MoS_2_ NS and AuNRs in MoS_2_@AuNR nanocomposites. For photothermal therapy, a minimum temperature of 50 °C was able to destroy *E. coli* [35]. The temperature of the MoS_2_@AuNR solution reached 50 °C with NIR laser illumination for 2 min. Therefore, an irradiation time of 2 min was set for MoS_2_@AuNRs in the following application of photothermal therapy.

### 3.6. ROS Generation by MoS_2_@AuNRs

**Figure 6:**
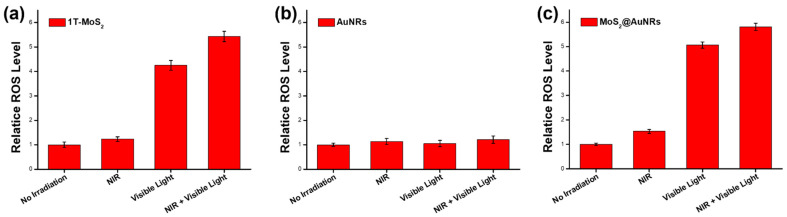
Relative reactive oxygen species (ROS) levels of (**a**) metallic molybdenum disulfide (1T-MoS_2_) nanosheets (NSs), (**b**) gold nanorods (AuNRs), and (**c**) MoS_2_@AuNRs with no light irradiation and with near infrared (NIR) laser, visible light, and both NIR laser and visible light irradiation. All data are presented as the mean ± SD, *n* = 3 per group.

To examine photodynamic performance, the ROS generated by 1T-MoS_2_ NSs, AuNRs, and MoS_2_@AuNRs were investigated under NIR laser and visible light irradiation based on the H2DCFDA assay. As shown in Figure 6, the ROS levels of 1T-MoS_2_ NSs, AuNRs, and MoS_2_@AuNRs without irradiation were set to 1.0. As shown in Figure 6a, the relative ROS levels of 1T-MoS_2_ NSs with NIR laser (2 min), visible light (1 min), and both NIR laser (2 min) and visible light (1 min) irradiation were respectively 1.23-, 4.25-, and 5.43-fold higher compared to that of no irradiation. The results could be because metallic 1T-MoS_2_ NSs with photocatalytic activity induced the ROS generation under visible light [46]. However, for the AuNRs, with NIR laser, visible light, and both NIR laser and visible light irradiation, there were no significant increases in ROS production, as shown in Figure 6b. Most importantly, the relative ROS levels of MoS_2_@AuNRs with NIR laser, visible light, and both NIR laser and visible light irradiation were 1.53-, 5.06-, and 5.81-fold higher, respectively, compared to that of no irradiation as shown in Figure 6c. With NIR laser irradiation, there was no significant production of ROS from MoS_2_@AuNRs because very few light-induced ROS were generated by the 1T-MoS_2_ NSs and AuNRs. However, with visible light irradiation, remarkable light-induced ROS production from MoS_2_@AuNRs was measured due to the visible-light activity of the 1T-MoS_2_ NSs. Furthermore, with both NIR laser and visible light irradiation, the light-induced ROS production from the MoS_2_@AuNRs did not noticeably increase compared to that of MoS_2_@AuNRs with only visible light irradiation. The reason could be the light-induced ROS production from MoS_2_@AuNRs mainly being contributed by the visible-light activity of the 1T-MoS_2_ NSs in the MoS_2_@AuNRs.

### 3.7. Evaluation of Photothermal Therapy and Photodynamic Therapy of MoS_2_@AuNRs

**Figure 7:**
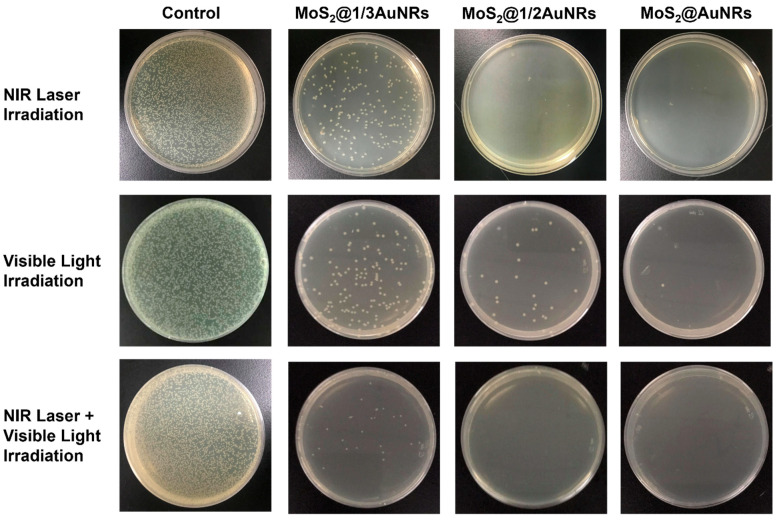
Photographs of the growth of *Escherichia coli* incubated with sterilized water (control) and molybdenum disulfide (MoS_2_)@1/3 gold nanorods (AuNRs), MoS_2_@1/2AuNRs, and MoS_2_@AuNRs on LB agar plates after NIR laser, visible light, or both NIR laser and visible light irradiation. The antibacterial rate of the control was set to 0% for each type of irradiation.

To evaluate the performance of photothermal therapy and photodynamic therapy, the light-induced bactericidal activities of MoS_2_@1/3AuNRs, MoS_2_@1/2AuNRs, and MoS_2_@AuNRs were investigated by agar plate counts. As shown in Figure 7, with NIR laser irradiation for 2 min, bacterial reductions by MoS_2_@1/3AuNRs, MoS_2_@1/2AuNRs, and MoS_2_@AuNRs were 84.4%, 97.5%, and 99.0%, respectively. The results indicated that an increase in AuNRs decorating 1T-MoS_2_ NSs improved the photothermal effect of the MoS_2_@1/3AuNR, MoS_2_@1/2AuNR, and MoS_2_@AuNR nanocomposites. Therefore, with the highest concentration of AuNRs, MoS_2_@AuNRs exhibited the best photothermal therapy compared to MoS_2_@1/3AuNRs and MoS_2_@1/2AuNRs. With visible light irradiation (1 min), the antibacterial rates of MoS_2_@1/3AuNRs, MoS_2_@1/2AuNRs, and MoS_2_@AuNRs were 83.8%, 93.3%, and 98.5%, respectively. Under visible light irradiation, the antibacterial activities of MoS_2_@1/3AuNRs, MoS_2_@1/2AuNRs, and MoS_2_@AuNRs were attributed to their light-induced ROS generation. Most importantly, to investigate the synergistic effect of photothermal therapy and photodynamic therapy, MoS_2_@1/3AuNRs, MoS_2_@1/2AuNRs, and MoS_2_@AuNRs were sequentially irradiated with NIR laser and visible light. After irradiation with NIR laser and visible light, the bactericidal efficiencies of MoS_2_@1/3AuNRs, MoS_2_@1/2AuNRs, and MoS_2_@AuNRs were 94.5%, 100%, and 100%, respectively. With the combination of NIR laser and visible light irradiation, significant increases in bactericidal efficiencies were demonstrated compared to irradiation by only the NIR laser or visible light. Overall, with the decoration of the photothermal agent of AuNRs onto the photodynamic agent of 1T-MoS_2_ NSs, the synergistic effect of photothermal therapy and photodynamic therapy in the nanocomposites of MoS_2_@1/3AuNRs, MoS_2_@1/2AuNRs, and MoS_2_@AuNRs was successfully demonstrated for an antibacterial application.

## 4. Conclusions

In conclusion, the AuNR photothermal agent was successfully decorated onto the 1T-MoS_2_ NS photodynamic agent by electrostatic adsorption to form MoS_2_@AuNRs. The optical and structural properties of MoS_2_@AuNRs were further demonstrated by UV–Vis spectroscopy, TEM, zeta potential, FTIR, and XRD. The photothermal performance validated that the final temperatures of MoS_2_@AuNRs, MoS_2_@1/2AuNRs, and MoS_2_@1/3AuNRs reached 66.7, 62.9, and 59.5 °C, respectively, after 808 nm NIR laser irradiation for 10 min. For the photodynamic performance, light-induced ROS from MoS_2_@AuNRs were generated mainly by the visible-light activity of 1T-MoS_2_ NSs in MoS_2_@AuNRs. With the combination of the AuNR photothermal agent and the 1T-MoS_2_ NS photodynamic agent, the MoS_2_@1/3AuNR, MoS_2_@1/2AuNR, and MoS_2_@AuNR nanocomposites exhibited synergistic effects of photothermal therapy and photodynamic therapy against bacteria. This work confirmed that MoS_2_@AuNR nanocomposites with a superior synergistic effect of photothermal therapy and photodynamic therapy could be a promising light-activated antibacterial agent in food safety, water sterilization, antibacterial paint, and medical therapy in the near future.

## Data Availability

The data presented in this study are available on request from the corresponding author.
